# Supplementary material for: Deletion of wfs1 Impairs Oligodendrocyte Precursor Cells Dorsal Distribution and Myelination Through the wfs1-hmgcs1 Axis in Zebrafish
Source: Biology (Basel). 2026 Aug 16;15(16):1402. doi: 10.3390/biology15161402 (PMC13510151; doi:10.3390/biology15161402)
Supplement: Supplementary file 1 [file biology-15-01402-s001.zip › File S1-Transcriptome sequencing data and differentially expressed genes (DEGs) in wfs1-- zebrafish at 5 dpf/2.KEGG/wfs1KOvsWT/GSEA/WT/CALCIUM_SIGNALING_PATHWAY/CALCIUM_SIGNALING_PATHWAY(DRE04020).html]

Details for gene set CALCIUM\_SIGNALING\_PATHWAY(DRE04020)[GSEA]

|  || Dataset | deg.sample |
| Phenotype | sample.cls |
| Upregulated in class | WT |
| GeneSet | CALCIUM\_SIGNALING\_PATHWAY(DRE04020) |
| Enrichment Score (ES) | -0.22612691 |
| Normalized Enrichment Score (NES) | -1.1241972 |
| Nominal p-value | 0.18533605 |
| FDR q-value | 0.51600647 |
| FWER p-Value | 1.0 |
Table: GSEA Results Summary

  

Fig 1: Enrichment plot: CALCIUM\_SIGNALING\_PATHWAY(DRE04020)      
 Profile of the Running ES Score & Positions of GeneSet Members on the Rank Ordered List

  

| PROBE | DESCRIPTION (from dataset) | GENE SYMBOL | GENE\_TITLE | RANK IN GENE LIST | RANK METRIC SCORE | RUNNING ES | CORE ENRICHMENT || 1 | 368717 | itpka |  |  | 192 | 4.239 | 0.0099 | No |
| 2 | 393186\_1 | zgc:56235 |  |  | 453 | 3.118 | 0.0134 | No |
| 3 | 544664\_1 | erbb3b |  |  | 580 | 2.789 | 0.0200 | No |
| 4 | 58147 | p2rx3a |  |  | 652 | 2.679 | 0.0278 | No |
| 5 | 100006675 | prkcba |  |  | 711 | 2.563 | 0.0357 | No |
| 6 | 494456 | fgf23 |  |  | 754 | 2.463 | 0.0436 | No |
| 7 | 336121 | calm2a |  |  | 870 | 2.292 | 0.0487 | No |
| 8 | 796490\_2 | pdgfbb |  |  | 882 | 2.259 | 0.0568 | No |
| 9 | 558525\_1 | atp2b3b |  |  | 981 | 2.114 | 0.0617 | No |
| 10 | 58133 | ngfb |  |  | 1219 | 1.881 | 0.0613 | No |
| 11 | 568726 | adcy7 |  |  | 1220 | 1.881 | 0.0684 | No |
| 12 | 101886891 | LOC101886891 |  |  | 1249 | 1.852 | 0.0745 | No |
| 13 | 103909601 | LOC103909601 |  |  | 1276 | 1.830 | 0.0806 | No |
| 14 | 393940 | atp2a2a |  |  | 1653 | 1.567 | 0.0746 | No |
| 15 | 557258 | ptk2ba |  |  | 1750 | 1.505 | 0.0772 | No |
| 16 | 555941 | cacna1ba |  |  | 1894 | 1.415 | 0.0780 | No |
| 17 | 493181 | fgf7 |  |  | 1929 | 1.397 | 0.0822 | No |
| 18 | 564017 | tacr1b |  |  | 1970 | 1.379 | 0.0861 | No |
| 19 | 563724 | adcy2a |  |  | 2111 | 1.321 | 0.0867 | No |
| 20 | 560875 | cacna1ha |  |  | 2236 | 1.271 | 0.0875 | No |
| 21 | 100003539\_1 | tacr1a |  |  | 2253 | 1.265 | 0.0918 | No |
| 22 | 100137117\_1 | si:dkey-1h24.2 |  |  | 2324 | 1.240 | 0.0943 | No |
| 23 | 794365 | si:dkey-163m14.7 |  |  | 2337 | 1.233 | 0.0985 | No |
| 24 | 559806 | htr2ab |  |  | 2342 | 1.232 | 0.1031 | No |
| 25 | 560564 | pdgfc |  |  | 2476 | 1.180 | 0.1033 | No |
| 26 | 555548 | pln |  |  | 2594 | 1.146 | 0.1039 | No |
| 27 | 557499 | slc8a4b |  |  | 2704 | 1.110 | 0.1046 | No |
| 28 | 100005590 | hrh2b |  |  | 2837 | 1.074 | 0.1045 | No |
| 29 | 561635 | mylk3 |  |  | 2947 | 1.051 | 0.1050 | No |
| 30 | 559529 | pdgfrb |  |  | 3029 | 1.028 | 0.1063 | No |
| 31 | 100535026 | ltb4r2a |  |  | 3060 | 1.022 | 0.1092 | No |
| 32 | 558477 | f2r |  |  | 3107 | 1.011 | 0.1116 | No |
| 33 | 553296 | hrc |  |  | 3174 | 0.998 | 0.1133 | No |
| 34 | 103909601\_1 | LOC103909601 |  |  | 3250 | 0.982 | 0.1146 | No |
| 35 | 402920 | lhcgr |  |  | 3255 | 0.981 | 0.1182 | No |
| 36 | 567611 | itpr1a |  |  | 3360 | 0.962 | 0.1185 | No |
| 37 | 565241 | ppp3ccb |  |  | 3535 | 0.922 | 0.1164 | No |
| 38 | 570493 | grin2ab |  |  | 3683 | 0.896 | 0.1151 | No |
| 39 | 557194\_1 | adrb1 |  |  | 3690 | 0.895 | 0.1183 | No |
| 40 | 494489 | atp2a1l |  |  | 3699 | 0.892 | 0.1215 | No |
| 41 | 492486 | adra1bb |  |  | 3825 | 0.864 | 0.1207 | No |
| 42 | 335654 | camk1ga |  |  | 3834 | 0.862 | 0.1237 | No |
| 43 | 570435 | fgf4 |  |  | 3855 | 0.858 | 0.1264 | No |
| 44 | 494454\_1 | fgf5 |  |  | 3873 | 0.854 | 0.1290 | No |
| 45 | 100334891 | LOC100334891 |  |  | 3900 | 0.848 | 0.1314 | No |
| 46 | 447814 | ppp3r1b |  |  | 3939 | 0.841 | 0.1334 | No |
| 47 | 569706\_1 | phkb |  |  | 3949 | 0.839 | 0.1363 | No |
| 48 | 569480 | slc8a2a |  |  | 4033 | 0.822 | 0.1367 | No |
| 49 | 368199 | bdkrb1 |  |  | 4038 | 0.822 | 0.1397 | No |
| 50 | 403311 | cacna1db |  |  | 4082 | 0.814 | 0.1414 | No |
| 51 | 30512 | ret |  |  | 4208 | 0.792 | 0.1404 | No |
| 52 | 100005700 | stim1b |  |  | 4287 | 0.778 | 0.1409 | No |
| 53 | 114550 | ednraa |  |  | 4376 | 0.765 | 0.1410 | No |
| 54 | 559201 | tacr3l |  |  | 4448 | 0.752 | 0.1416 | No |
| 55 | 100037315 | adrb2b |  |  | 4505 | 0.744 | 0.1426 | No |
| 56 | 566370\_1 | slc25a6 |  |  | 4580 | 0.736 | 0.1430 | No |
| 57 | 445226 | casq1a |  |  | 4633 | 0.730 | 0.1441 | No |
| 58 | 322720 | itpkcb |  |  | 4736 | 0.713 | 0.1436 | No |
| 59 | 792519 | adrb3b |  |  | 4793 | 0.706 | 0.1445 | No |
| 60 | 100537538 | LOC100537538 |  |  | 4897 | 0.694 | 0.1438 | No |
| 61 | 565379 | phkg1a |  |  | 4932 | 0.689 | 0.1454 | No |
| 62 | 58129\_1 | fgfr3 |  |  | 4965 | 0.686 | 0.1469 | No |
| 63 | 564719 | adcy3b |  |  | 4985 | 0.683 | 0.1489 | No |
| 64 | 571591 | avpr1aa |  |  | 5008 | 0.680 | 0.1508 | No |
| 65 | 494455 | fgf6b |  |  | 5163 | 0.660 | 0.1484 | No |
| 66 | 562059 | cacna1aa |  |  | 5382 | 0.641 | 0.1439 | No |
| 67 | 100001636 | drd6b |  |  | 5401 | 0.640 | 0.1457 | No |
| 68 | 100002448 | mst1ra |  |  | 5412 | 0.640 | 0.1478 | No |
| 69 | 394199 | chrna7 |  |  | 5483 | 0.635 | 0.1480 | No |
| 70 | 100150664 | met |  |  | 5592 | 0.627 | 0.1469 | No |
| 71 | 334582 | vdac1 |  |  | 5612 | 0.625 | 0.1487 | No |
| 72 | 566141 | si:ch211-39a7.1 |  |  | 5685 | 0.619 | 0.1487 | No |
| 73 | 561747 | plcg2 |  |  | 5761 | 0.610 | 0.1486 | No |
| 74 | 678512 | figf |  |  | 5864 | 0.601 | 0.1477 | No |
| 75 | 327067 | slc25a4 |  |  | 5868 | 0.600 | 0.1498 | No |
| 76 | 386635 | erbb3a |  |  | 5954 | 0.592 | 0.1494 | No |
| 77 | 641328 | ppifb |  |  | 6071 | 0.580 | 0.1479 | No |
| 78 | 100007677 | erbb4a |  |  | 6125 | 0.574 | 0.1484 | No |
| 79 | 101882850 | htr4 |  |  | 6158 | 0.572 | 0.1495 | No |
| 80 | 387301\_1 | p2rx4b |  |  | 6184 | 0.570 | 0.1509 | No |
| 81 | 393998 | prkacba |  |  | 6234 | 0.564 | 0.1514 | No |
| 82 | 100331465 | LOC100331465 |  |  | 6276 | 0.559 | 0.1523 | No |
| 83 | 798222 | camk2b |  |  | 6413 | 0.544 | 0.1500 | No |
| 84 | 30705 | fgfr1a |  |  | 6434 | 0.542 | 0.1514 | No |
| 85 | 100334881 | stim2a |  |  | 6452 | 0.541 | 0.1529 | No |
| 86 | 100149482 | LOC100149482 |  |  | 6594 | 0.528 | 0.1504 | No |
| 87 | 565241\_1 | ppp3ccb |  |  | 6607 | 0.527 | 0.1520 | No |
| 88 | 30682 | vegfaa |  |  | 6618 | 0.526 | 0.1537 | No |
| 89 | 568463\_2 | ryr2b |  |  | 6731 | 0.516 | 0.1521 | No |
| 90 | 560561 | slc8a3 |  |  | 6854 | 0.504 | 0.1501 | No |
| 91 | 100333455 | agtr1b |  |  | 6916 | 0.499 | 0.1501 | No |
| 92 | 100536162 | pde1cb |  |  | 6926 | 0.498 | 0.1517 | No |
| 93 | 493613 | gna11b |  |  | 7043 | 0.488 | 0.1498 | No |
| 94 | 564465 | pde1a |  |  | 7073 | 0.485 | 0.1507 | No |
| 95 | 777614 | tpcn2 |  |  | 7081 | 0.485 | 0.1523 | No |
| 96 | 30560 | fgf6a |  |  | 7105 | 0.482 | 0.1534 | No |
| 97 | 100334116 | LOC100334116 |  |  | 7155 | 0.477 | 0.1537 | No |
| 98 | 114447 | cxcr4b |  |  | 7160 | 0.476 | 0.1553 | No |
| 99 | 556360 | stim1a |  |  | 7189 | 0.475 | 0.1562 | No |
| 100 | 557926\_2 | ppp3cca |  |  | 7193 | 0.474 | 0.1579 | No |
| 101 | 101885082 | gdnfb |  |  | 7227 | 0.472 | 0.1587 | No |
| 102 | 259258\_1 | p2rx4a |  |  | 7259 | 0.469 | 0.1595 | No |
| 103 | 559964\_2 | cacna1fb |  |  | 7527 | 0.453 | 0.1527 | No |
| 104 | 793359\_1 | camk2g2 |  |  | 7602 | 0.447 | 0.1520 | No |
| 105 | 100333270 | LOC100333270 |  |  | 7751 | 0.439 | 0.1490 | No |
| 106 | 403049 | vegfc |  |  | 7924 | 0.429 | 0.1451 | No |
| 107 | 100003342 | il12rb2l |  |  | 7996 | 0.424 | 0.1445 | No |
| 108 | 405866 | ednrab |  |  | 8036 | 0.421 | 0.1448 | No |
| 109 | 562585 | itpr2 |  |  | 8204 | 0.415 | 0.1411 | No |
| 110 | 562796\_1 | cacna1bb |  |  | 8318 | 0.411 | 0.1390 | No |
| 111 | 100537531 | si:ch73-380n15.2 |  |  | 8330 | 0.411 | 0.1402 | No |
| 112 | 794026 | camk1b |  |  | 8440 | 0.407 | 0.1383 | No |
| 113 | 100034592 | gna15.3 |  |  | 8475 | 0.405 | 0.1388 | No |
| 114 | 767790 | pdgfab |  |  | 8507 | 0.402 | 0.1393 | No |
| 115 | 402892\_1 | orai1b |  |  | 8536 | 0.400 | 0.1399 | No |
| 116 | 557926 | ppp3cca |  |  | 8563 | 0.398 | 0.1406 | No |
| 117 | 572143 | htr7c |  |  | 8694 | 0.388 | 0.1379 | No |
| 118 | 568288 | plce1 |  |  | 8774 | 0.383 | 0.1369 | No |
| 119 | 566118 | adora2ab |  |  | 8867 | 0.377 | 0.1353 | No |
| 120 | 793708 | cacna1fa |  |  | 8992 | 0.368 | 0.1328 | No |
| 121 | 564945 | ptger1b |  |  | 9008 | 0.367 | 0.1337 | No |
| 122 | 566351 | cacna1i |  |  | 9017 | 0.366 | 0.1348 | No |
| 123 | 100332913 | grm5b |  |  | 9058 | 0.363 | 0.1349 | No |
| 124 | 767745 | grin1a |  |  | 9072 | 0.362 | 0.1359 | No |
| 125 | 560100 | adora2b |  |  | 9076 | 0.362 | 0.1372 | No |
| 126 | 567498 | LOC567498 |  |  | 9176 | 0.354 | 0.1354 | No |
| 127 | 559630 | fgf20a |  |  | 9306 | 0.346 | 0.1326 | No |
| 128 | 555939 | avpr1ab |  |  | 9380 | 0.342 | 0.1315 | No |
| 129 | 405791 | cacna1sb |  |  | 9453 | 0.337 | 0.1305 | No |
| 130 | 387298\_1 | p2rx7 |  |  | 9469 | 0.336 | 0.1313 | No |
| 131 | 100038775 | htr5aa |  |  | 9489 | 0.335 | 0.1320 | No |
| 132 | 571825 | adcy3a |  |  | 9521 | 0.332 | 0.1322 | No |
| 133 | 387301\_2 | p2rx4b |  |  | 9555 | 0.330 | 0.1324 | No |
| 134 | 793359 | camk2g2 |  |  | 9631 | 0.325 | 0.1313 | No |
| 135 | 797199\_1 | slc8a1b |  |  | 9790 | 0.316 | 0.1274 | No |
| 136 | 796298 | oxtrl |  |  | 9803 | 0.316 | 0.1283 | No |
| 137 | 492467 | gnal |  |  | 10100 | 0.296 | 0.1200 | No |
| 138 | 791176 | camk1db |  |  | 10201 | 0.290 | 0.1179 | No |
| 139 | 245950 | ptk2bb |  |  | 10211 | 0.290 | 0.1187 | No |
| 140 | 387296 | p2rx1 |  |  | 10248 | 0.288 | 0.1186 | No |
| 141 | 100535718 | pdgfd |  |  | 10271 | 0.286 | 0.1190 | No |
| 142 | 568463 | ryr2b |  |  | 10274 | 0.286 | 0.1200 | No |
| 143 | 768182 | mcu |  |  | 10356 | 0.281 | 0.1185 | No |
| 144 | 569836 | erbb4b |  |  | 10364 | 0.281 | 0.1194 | No |
| 145 | 394123 | mcoln2 |  |  | 10373 | 0.281 | 0.1202 | No |
| 146 | 386856 | pdgfra |  |  | 10436 | 0.277 | 0.1192 | No |
| 147 | 554230 | kdr |  |  | 10614 | 0.265 | 0.1146 | No |
| 148 | 570240 | ntrk2b |  |  | 10759 | 0.257 | 0.1110 | No |
| 149 | 100124597 | zgc:171965 |  |  | 10778 | 0.256 | 0.1114 | No |
| 150 | 497384 | prkca |  |  | 10900 | 0.249 | 0.1085 | No |
| 151 | 100333538 | LOC100333538 |  |  | 11027 | 0.242 | 0.1054 | No |
| 152 | 569528 | cacna1ab |  |  | 11030 | 0.242 | 0.1063 | No |
| 153 | 560410 | si:ch211-132f19.7 |  |  | 11057 | 0.241 | 0.1063 | No |
| 154 | 553978 | chrm5a |  |  | 11082 | 0.240 | 0.1065 | No |
| 155 | 565669 | plcd4a |  |  | 11125 | 0.238 | 0.1060 | No |
| 156 | 407700 | plcb4 |  |  | 11151 | 0.236 | 0.1061 | No |
| 157 | 100149080 | LOC100149080 |  |  | 11197 | 0.233 | 0.1056 | No |
| 158 | 563904 | cacna1g |  |  | 11395 | 0.222 | 0.1002 | No |
| 159 | 555443 | plcd1a |  |  | 11457 | 0.219 | 0.0991 | No |
| 160 | 561491 | chrm5b |  |  | 11490 | 0.217 | 0.0989 | No |
| 161 | 563019 | itpkb |  |  | 11517 | 0.216 | 0.0988 | No |
| 162 | 562569 | plcd3b |  |  | 11668 | 0.208 | 0.0949 | No |
| 163 | 567534 | tpcn1 |  |  | 11680 | 0.207 | 0.0953 | No |
| 164 | 555516\_1 | chrm2b |  |  | 11717 | 0.205 | 0.0949 | No |
| 165 | 100334819 | mcoln3a |  |  | 11736 | 0.204 | 0.0951 | No |
| 166 | 393953 | prkcbb |  |  | 11901 | 0.196 | 0.0906 | No |
| 167 | 30121 | flt4 |  |  | 11941 | 0.193 | 0.0901 | No |
| 168 | 560719 | si:dkey-206f10.1 |  |  | 11948 | 0.193 | 0.0907 | No |
| 169 | 101885162 | si:ch73-383l1.1 |  |  | 12003 | 0.191 | 0.0897 | No |
| 170 | 735302 | hrh1 |  |  | 12088 | 0.186 | 0.0877 | No |
| 171 | 100003514 | prkcg |  |  | 12176 | 0.181 | 0.0856 | No |
| 172 | 100537296 | ltb4r2b |  |  | 12192 | 0.179 | 0.0858 | No |
| 173 | 445232 | gna15.1 |  |  | 12197 | 0.179 | 0.0864 | No |
| 174 | 567905 | fgfr1b |  |  | 12317 | 0.173 | 0.0832 | No |
| 175 | 798498 | adra1aa |  |  | 12347 | 0.171 | 0.0830 | No |
| 176 | 792634 | drd1a |  |  | 12455 | 0.167 | 0.0802 | No |
| 177 | 100334963 | cysltr3 |  |  | 12474 | 0.165 | 0.0802 | No |
| 178 | 557353 | gnas |  |  | 12479 | 0.165 | 0.0807 | No |
| 179 | 103909114 | LOC103909114 |  |  | 12547 | 0.162 | 0.0792 | No |
| 180 | 359830\_1 | fgf10a |  |  | 12579 | 0.161 | 0.0788 | No |
| 181 | 378746 | atp2b1a |  |  | 12737 | 0.152 | 0.0744 | No |
| 182 | 796537 | kdrl |  |  | 12763 | 0.151 | 0.0742 | No |
| 183 | 102216340 | tbxa2r |  |  | 12832 | 0.148 | 0.0726 | No |
| 184 | 259260 | mst1 |  |  | 12853 | 0.147 | 0.0725 | No |
| 185 | 562919 | itpkca |  |  | 13039 | 0.138 | 0.0672 | No |
| 186 | 140747 | cxcr4a |  |  | 13072 | 0.136 | 0.0667 | No |
| 187 | 559315 | ptger1a |  |  | 13115 | 0.135 | 0.0658 | No |
| 188 | 562064 | camk2b1 |  |  | 13117 | 0.134 | 0.0663 | No |
| 189 | 407737 | fgf17 |  |  | 13146 | 0.133 | 0.0659 | No |
| 190 | 570108 | gnaq |  |  | 13257 | 0.128 | 0.0629 | No |
| 191 | 436745 | atp2b3a |  |  | 13270 | 0.127 | 0.0630 | No |
| 192 | 558685\_1 | mylk2 |  |  | 13378 | 0.122 | 0.0601 | No |
| 193 | 100004739 | cysltr2a |  |  | 13552 | 0.113 | 0.0550 | No |
| 194 | 797212 | LOC797212 |  |  | 13682 | 0.107 | 0.0513 | No |
| 195 | 557902 | adcy2b |  |  | 13739 | 0.105 | 0.0499 | No |
| 196 | 566845 | mylk4a |  |  | 13862 | 0.099 | 0.0464 | No |
| 197 | 563147 | prkacbb |  |  | 13940 | 0.095 | 0.0443 | No |
| 198 | 101884544 | LOC101884544 |  |  | 14218 | 0.081 | 0.0358 | No |
| 199 | 100149388 | itpr1b |  |  | 14261 | 0.080 | 0.0348 | No |
| 200 | 557026 | adcy1a |  |  | 14359 | 0.076 | 0.0320 | No |
| 201 | 794575 | agtr1a |  |  | 14442 | 0.072 | 0.0296 | No |
| 202 | 58129 | fgfr3 |  |  | 14448 | 0.072 | 0.0298 | No |
| 203 | 564064 | prkacaa |  |  | 14469 | 0.071 | 0.0294 | No |
| 204 | 563111 | cysltr2b |  |  | 14475 | 0.070 | 0.0295 | No |
| 205 | 100001532 | plcb3 |  |  | 14510 | 0.069 | 0.0287 | No |
| 206 | 724048 | fgf21 |  |  | 14591 | 0.065 | 0.0264 | No |
| 207 | 100038774 | si:dkey-251i10.1 |  |  | 14670 | 0.062 | 0.0241 | No |
| 208 | 359829 | fgf18a |  |  | 14982 | 0.047 | 0.0144 | No |
| 209 | 794658 | chrm1b |  |  | 15086 | 0.042 | 0.0113 | No |
| 210 | 100005675 | grin1b |  |  | 15101 | 0.041 | 0.0110 | No |
| 211 | 100331840 | sphk1 |  |  | 15103 | 0.041 | 0.0111 | No |
| 212 | 30442 | ednrba |  |  | 15136 | 0.040 | 0.0103 | No |
| 213 | 493781 | hgfa |  |  | 15196 | 0.037 | 0.0085 | No |
| 214 | 447887 | ppifa |  |  | 15238 | 0.035 | 0.0074 | No |
| 215 | 100149100 | adra1ba |  |  | 15262 | 0.034 | 0.0068 | No |
| 216 | 557259 | adra1ab |  |  | 15287 | 0.033 | 0.0061 | No |
| 217 | 321808 | calm3b |  |  | 15342 | 0.031 | 0.0045 | No |
| 218 | 352940 | fgfr2 |  |  | 15408 | 0.027 | 0.0025 | No |
| 219 | 563654 | nos2b |  |  | 15462 | 0.024 | 0.0010 | No |
| 220 | 568668 | ntrk3a |  |  | 15467 | 0.024 | 0.0009 | No |
| 221 | 497639 | fgf18b |  |  | 15472 | 0.024 | 0.0009 | No |
| 222 | 751784 | htr2b |  |  | 15535 | 0.020 | -0.0010 | No |
| 223 | 561701\_1 | adora2aa |  |  | 15790 | 0.007 | -0.0091 | No |
| 224 | 563953\_1 | gna11a |  |  | 15848 | 0.005 | -0.0109 | No |
| 225 | 387299 | p2rx8 |  |  | 15976 | -0.000 | -0.0149 | No |
| 226 | 798565 | ryr1a |  |  | 15984 | -0.001 | -0.0151 | No |
| 227 | 373867 | plcg1 |  |  | 16057 | -0.004 | -0.0174 | No |
| 228 | 170581 | cacna1c |  |  | 16091 | -0.005 | -0.0184 | No |
| 229 | 556431 | trhr2 |  |  | 16192 | -0.008 | -0.0216 | No |
| 230 | 563297 | grin2aa |  |  | 16270 | -0.010 | -0.0240 | No |
| 231 | 100333648 | grin2cb |  |  | 16330 | -0.012 | -0.0258 | No |
| 232 | 359831 | fgf24 |  |  | 16353 | -0.012 | -0.0265 | No |
| 233 | 562640 | si:dkey-28b4.8 |  |  | 16374 | -0.013 | -0.0271 | No |
| 234 | 568126 | drd1b |  |  | 16404 | -0.014 | -0.0279 | No |
| 235 | 553675\_1 | cysltr1 |  |  | 16416 | -0.015 | -0.0282 | No |
| 236 | 378478 | egfra |  |  | 16429 | -0.015 | -0.0285 | No |
| 237 | 794026\_1 | camk1b |  |  | 16459 | -0.016 | -0.0294 | No |
| 238 | 568463\_1 | ryr2b |  |  | 16528 | -0.018 | -0.0315 | No |
| 239 | 561190 | atp2b1b |  |  | 16586 | -0.020 | -0.0332 | No |
| 240 | 799067 | LOC799067 |  |  | 16617 | -0.021 | -0.0341 | No |
| 241 | 58082 | tnnc2 |  |  | 16621 | -0.021 | -0.0341 | No |
| 242 | 561350 | ryr3 |  |  | 16893 | -0.030 | -0.0426 | No |
| 243 | 558154 | vegfab |  |  | 16935 | -0.032 | -0.0438 | No |
| 244 | 792708 | chrm1a |  |  | 17029 | -0.034 | -0.0467 | No |
| 245 | 100536080 | htr7 |  |  | 17053 | -0.035 | -0.0473 | No |
| 246 | 100002627 | ptafr |  |  | 17086 | -0.036 | -0.0481 | No |
| 247 | 100004938 | si:dkey-63b1.1 |  |  | 17158 | -0.039 | -0.0503 | No |
| 248 | 445076 | prkacab |  |  | 17201 | -0.041 | -0.0514 | No |
| 249 | 569499 | adcy1b |  |  | 17365 | -0.048 | -0.0564 | No |
| 250 | 100000160 | fgfr4 |  |  | 17448 | -0.051 | -0.0588 | No |
| 251 | 100002038\_1 | si:rp71-17i16.4 |  |  | 17829 | -0.069 | -0.0707 | No |
| 252 | 797199 | slc8a1b |  |  | 17863 | -0.070 | -0.0715 | No |
| 253 | 368245 | fgf19 |  |  | 17864 | -0.070 | -0.0712 | No |
| 254 | 30270 | ngfa |  |  | 17981 | -0.075 | -0.0746 | No |
| 255 | 406529 | vdac3 |  |  | 17989 | -0.075 | -0.0745 | No |
| 256 | 767790\_1 | pdgfab |  |  | 18004 | -0.076 | -0.0747 | No |
| 257 | 568671 | atp2a3 |  |  | 18013 | -0.076 | -0.0747 | No |
| 258 | 100001530 | oxtr |  |  | 18039 | -0.078 | -0.0752 | No |
| 259 | 555945 | camk1a |  |  | 18072 | -0.080 | -0.0759 | No |
| 260 | 103909426 | LOC103909426 |  |  | 18207 | -0.085 | -0.0798 | No |
| 261 | 100537531\_1 | si:ch73-380n15.2 |  |  | 18213 | -0.086 | -0.0797 | No |
| 262 | 100001113 | mcoln3b |  |  | 18498 | -0.099 | -0.0883 | No |
| 263 | 100333702 | adcy9 |  |  | 18501 | -0.099 | -0.0880 | No |
| 264 | 449864 | grin2da |  |  | 18535 | -0.100 | -0.0887 | No |
| 265 | 101882451 | LOC101882451 |  |  | 18545 | -0.101 | -0.0886 | No |
| 266 | 569040 | plcd3a |  |  | 18746 | -0.111 | -0.0945 | No |
| 267 | 100005852\_1 | fgf22 |  |  | 18778 | -0.112 | -0.0951 | No |
| 268 | 100149598 | chrm3b |  |  | 18785 | -0.112 | -0.0949 | No |
| 269 | 565838 | adrb2a |  |  | 18862 | -0.116 | -0.0968 | No |
| 270 | 386966 | erbb2 |  |  | 18881 | -0.117 | -0.0970 | No |
| 271 | 368475 | htr5ab |  |  | 18905 | -0.118 | -0.0973 | No |
| 272 | 564274 | cacna1e |  |  | 19028 | -0.123 | -0.1007 | No |
| 273 | 100537531\_2 | si:ch73-380n15.2 |  |  | 19359 | -0.138 | -0.1106 | No |
| 274 | 352938 | chrm2a |  |  | 19449 | -0.142 | -0.1129 | No |
| 275 | 557194 | adrb1 |  |  | 19650 | -0.150 | -0.1187 | No |
| 276 | 798657\_1 | si:ch211-79m20.1 |  |  | 19845 | -0.159 | -0.1243 | No |
| 277 | 100003770 | mylk5 |  |  | 19866 | -0.160 | -0.1243 | No |
| 278 | 563953 | gna11a |  |  | 19873 | -0.160 | -0.1239 | No |
| 279 | 30546 | ntrk1 |  |  | 20060 | -0.169 | -0.1292 | No |
| 280 | 260440 | atp2a1 |  |  | 20102 | -0.171 | -0.1299 | No |
| 281 | 404231 | fgf2 |  |  | 20121 | -0.171 | -0.1298 | No |
| 282 | 406689 | mcoln1a |  |  | 20236 | -0.176 | -0.1327 | No |
| 283 | 791855 | camk2g1 |  |  | 20264 | -0.177 | -0.1329 | No |
| 284 | 192322 | calm2b |  |  | 20459 | -0.185 | -0.1384 | No |
| 285 | 569376 | plcb2 |  |  | 20503 | -0.188 | -0.1391 | No |
| 286 | 559236 | unm\_hu7910 |  |  | 20586 | -0.193 | -0.1409 | No |
| 287 | 794574 | ppp3ca |  |  | 20620 | -0.195 | -0.1412 | No |
| 288 | 336612 | ppiaa |  |  | 20653 | -0.196 | -0.1415 | No |
| 289 | 445226\_1 | casq1a |  |  | 20908 | -0.210 | -0.1488 | No |
| 290 | 368217 | calm1b |  |  | 21000 | -0.216 | -0.1509 | No |
| 291 | 393802 | camk1gb |  |  | 21070 | -0.219 | -0.1523 | No |
| 292 | 30538 | fgf8a |  |  | 21071 | -0.219 | -0.1514 | No |
| 293 | 550554 | ppp3cb |  |  | 21144 | -0.223 | -0.1529 | No |
| 294 | 30549 | fgf3 |  |  | 21330 | -0.233 | -0.1579 | No |
| 295 | 553675 | cysltr1 |  |  | 21580 | -0.245 | -0.1649 | No |
| 296 | 557430 | atp2b2 |  |  | 21585 | -0.245 | -0.1641 | No |
| 297 | 100144566 | camk1da |  |  | 21604 | -0.246 | -0.1637 | No |
| 298 | 569382 | trhrb |  |  | 21639 | -0.248 | -0.1639 | No |
| 299 | 65089 | fgf8b |  |  | 21694 | -0.251 | -0.1646 | No |
| 300 | 100333648\_1 | grin2cb |  |  | 21754 | -0.254 | -0.1655 | No |
| 301 | 192321 | slc25a5 |  |  | 21812 | -0.257 | -0.1664 | No |
| 302 | 101883995 | pde1ca |  |  | 21831 | -0.258 | -0.1660 | No |
| 303 | 568668\_1 | ntrk3a |  |  | 22137 | -0.275 | -0.1746 | No |
| 304 | 393937 | phkg2 |  |  | 22177 | -0.277 | -0.1748 | No |
| 305 | 322126 | vdac2 |  |  | 22259 | -0.282 | -0.1763 | No |
| 306 | 100334881\_1 | stim2a |  |  | 22313 | -0.285 | -0.1770 | No |
| 307 | 100150090 | sphk2 |  |  | 22316 | -0.285 | -0.1759 | No |
| 308 | 567702 | cacna1eb |  |  | 22385 | -0.289 | -0.1770 | No |
| 309 | 550436 | camk2a |  |  | 22386 | -0.289 | -0.1759 | No |
| 310 | 571679\_1 | chrm3a |  |  | 22392 | -0.289 | -0.1750 | No |
| 311 | 553489 | slc8a1a |  |  | 22439 | -0.292 | -0.1753 | No |
| 312 | 568265 | atp2a2b |  |  | 22467 | -0.294 | -0.1751 | No |
| 313 | 100148976\_1 | adcy8 |  |  | 22476 | -0.295 | -0.1742 | No |
| 314 | 114550\_1 | ednraa |  |  | 22532 | -0.298 | -0.1749 | No |
| 315 | 569510 | mylka |  |  | 22754 | -0.311 | -0.1807 | No |
| 316 | 407724 | gnal2 |  |  | 23024 | -0.329 | -0.1880 | No |
| 317 | 101884734 | LOC101884734 |  |  | 23075 | -0.333 | -0.1884 | No |
| 318 | 373879 | p2rx5 |  |  | 23105 | -0.335 | -0.1880 | No |
| 319 | 798577 | ntrk3b |  |  | 23106 | -0.335 | -0.1868 | No |
| 320 | 570245 | ryr1b |  |  | 23130 | -0.336 | -0.1862 | No |
| 321 | 567212 | slc8a2b |  |  | 23204 | -0.341 | -0.1872 | No |
| 322 | 555576 | grm1a |  |  | 23212 | -0.342 | -0.1862 | No |
| 323 | 387300 | p2rx3b |  |  | 23217 | -0.342 | -0.1850 | No |
| 324 | 100002125 | tacr2 |  |  | 23296 | -0.347 | -0.1862 | No |
| 325 | 406660 | calm1a |  |  | 23561 | -0.364 | -0.1932 | No |
| 326 | 58082\_1 | tnnc2 |  |  | 23591 | -0.366 | -0.1927 | No |
| 327 | 100037315\_1 | adrb2b |  |  | 23598 | -0.367 | -0.1915 | No |
| 328 | 79379 | gdnfa |  |  | 23663 | -0.371 | -0.1922 | No |
| 329 | 108179223 | LOC108179223 |  |  | 23726 | -0.375 | -0.1927 | No |
| 330 | 558878 | trhra |  |  | 23769 | -0.378 | -0.1926 | No |
| 331 | 795916 | mcoln1b |  |  | 23918 | -0.387 | -0.1959 | No |
| 332 | 799082 | si:dkey-163m14.2 |  |  | 23950 | -0.389 | -0.1954 | No |
| 333 | 570683 | cacna1sa |  |  | 24037 | -0.395 | -0.1966 | No |
| 334 | 556199 | phka2 |  |  | 24082 | -0.398 | -0.1965 | No |
| 335 | 572183 | phka1a |  |  | 24097 | -0.399 | -0.1955 | No |
| 336 | 562111\_2 | htr7a |  |  | 24129 | -0.401 | -0.1949 | No |
| 337 | 567611\_1 | itpr1a |  |  | 24145 | -0.403 | -0.1939 | No |
| 338 | 562625 | gna15.4 |  |  | 24353 | -0.415 | -0.1989 | No |
| 339 | 100150246\_1 | grm1b |  |  | 24426 | -0.416 | -0.1996 | No |
| 340 | 327379 | calm3a |  |  | 24599 | -0.422 | -0.2035 | No |
| 341 | 100002285 | drd7 |  |  | 24666 | -0.426 | -0.2040 | No |
| 342 | novel.939 | - |  |  | 24769 | -0.436 | -0.2056 | No |
| 343 | 403310 | cacna1da |  |  | 24910 | -0.442 | -0.2084 | No |
| 344 | 796490 | pdgfbb |  |  | 25120 | -0.455 | -0.2133 | No |
| 345 | 561701 | adora2aa |  |  | 25225 | -0.461 | -0.2149 | No |
| 346 | 101884360 | LOC101884360 |  |  | 25255 | -0.464 | -0.2140 | No |
| 347 | 100003535\_1 | ptger1c |  |  | 25343 | -0.470 | -0.2150 | No |
| 348 | 751792 | fgf10b |  |  | 25378 | -0.473 | -0.2143 | No |
| 349 | 100321746 | calml4b |  |  | 25428 | -0.478 | -0.2141 | No |
| 350 | novel.858 | - |  |  | 25529 | -0.487 | -0.2154 | No |
| 351 | 568614 | adra1d |  |  | 25558 | -0.490 | -0.2145 | No |
| 352 | 558248 | adrb3a |  |  | 25578 | -0.492 | -0.2132 | No |
| 353 | 100148976 | adcy8 |  |  | 25826 | -0.513 | -0.2191 | No |
| 354 | 436815 | camk2d2 |  |  | 25880 | -0.518 | -0.2188 | No |
| 355 | 114410 | casq2 |  |  | 25956 | -0.526 | -0.2192 | No |
| 356 | 550270 | camk4 |  |  | 26003 | -0.529 | -0.2187 | No |
| 357 | 60658 | nos1 |  |  | 26051 | -0.533 | -0.2182 | No |
| 358 | 563901 | stim2b |  |  | 26061 | -0.534 | -0.2165 | No |
| 359 | 568512 | slc8a4a |  |  | 26080 | -0.536 | -0.2150 | No |
| 360 | 558685 | mylk2 |  |  | 26141 | -0.541 | -0.2149 | No |
| 361 | 564770 | fgf20b |  |  | 26357 | -0.562 | -0.2196 | No |
| 362 | 100333967 | si:ch73-374l24.1 |  |  | 26564 | -0.584 | -0.2239 | Yes |
| 363 | 100144566\_1 | camk1da |  |  | 26613 | -0.589 | -0.2232 | Yes |
| 364 | 560151 | calml4a |  |  | 26620 | -0.590 | -0.2212 | Yes |
| 365 | 794278 | si:dkey-163m14.6 |  |  | 26637 | -0.591 | -0.2195 | Yes |
| 366 | 100000981 | htr2cl1 |  |  | 26669 | -0.595 | -0.2182 | Yes |
| 367 | 569344 | ntsr1 |  |  | 26699 | -0.599 | -0.2168 | Yes |
| 368 | 449707 | mylk4b |  |  | 26753 | -0.605 | -0.2163 | Yes |
| 369 | 373879\_1 | p2rx5 |  |  | 26833 | -0.613 | -0.2164 | Yes |
| 370 | 563567 | LOC563567 |  |  | 26854 | -0.614 | -0.2148 | Yes |
| 371 | 445208 | camk2d1 |  |  | 26863 | -0.615 | -0.2127 | Yes |
| 372 | 794666\_1 | itpr3 |  |  | 26950 | -0.625 | -0.2131 | Yes |
| 373 | 560808 | htr2aa |  |  | 26956 | -0.625 | -0.2109 | Yes |
| 374 | 565304\_2 | htr7b |  |  | 26993 | -0.628 | -0.2096 | Yes |
| 375 | 492576 | mylkb |  |  | 27047 | -0.634 | -0.2089 | Yes |
| 376 | 100003539 | tacr1a |  |  | 27082 | -0.638 | -0.2076 | Yes |
| 377 | 65090 | ntrk2a |  |  | 27134 | -0.642 | -0.2068 | Yes |
| 378 | 562796 | cacna1bb |  |  | 27140 | -0.642 | -0.2045 | Yes |
| 379 | 393733\_1 | fgf1a |  |  | 27202 | -0.645 | -0.2040 | Yes |
| 380 | 559964\_1 | cacna1fb |  |  | 27228 | -0.645 | -0.2024 | Yes |
| 381 | 101883614 | fgf9 |  |  | 27243 | -0.646 | -0.2004 | Yes |
| 382 | 794215 | LOC794215 |  |  | 27383 | -0.657 | -0.2023 | Yes |
| 383 | 497384\_1 | prkca |  |  | 27854 | -0.723 | -0.2145 | Yes |
| 384 | 556431\_1 | trhr2 |  |  | 27869 | -0.724 | -0.2123 | Yes |
| 385 | 387301 | p2rx4b |  |  | 27920 | -0.730 | -0.2111 | Yes |
| 386 | 544667\_1 | flt1 |  |  | 28017 | -0.744 | -0.2113 | Yes |
| 387 | 798657 | si:ch211-79m20.1 |  |  | 28060 | -0.751 | -0.2098 | Yes |
| 388 | 404036 | nos2a |  |  | 28157 | -0.765 | -0.2100 | Yes |
| 389 | 100126126 | ryr2a |  |  | 28171 | -0.768 | -0.2075 | Yes |
| 390 | 571679 | chrm3a |  |  | 28182 | -0.769 | -0.2049 | Yes |
| 391 | 569038 | cckar |  |  | 28194 | -0.771 | -0.2023 | Yes |
| 392 | 403045\_1 | egf |  |  | 28202 | -0.773 | -0.1996 | Yes |
| 393 | 100038781 | trdn |  |  | 28203 | -0.773 | -0.1967 | Yes |
| 394 | 554046 | phkg1b |  |  | 28450 | -0.811 | -0.2015 | Yes |
| 395 | 445297 | gna14 |  |  | 28514 | -0.824 | -0.2004 | Yes |
| 396 | 555945\_1 | camk1a |  |  | 28523 | -0.825 | -0.1975 | Yes |
| 397 | 100330960 | bdkrb2 |  |  | 28542 | -0.829 | -0.1950 | Yes |
| 398 | 555612 | orai2 |  |  | 28574 | -0.835 | -0.1928 | Yes |
| 399 | 563567\_1 | LOC563567 |  |  | 28582 | -0.836 | -0.1898 | Yes |
| 400 | 559089 | si:dkey-247m21.3 |  |  | 28628 | -0.843 | -0.1881 | Yes |
| 401 | 415175 | tnnc1b |  |  | 28699 | -0.858 | -0.1871 | Yes |
| 402 | 101883477 | LOC101883477 |  |  | 28848 | -0.885 | -0.1884 | Yes |
| 403 | 568406 | grm5a |  |  | 28855 | -0.888 | -0.1853 | Yes |
| 404 | 767757 | casq1b |  |  | 28934 | -0.905 | -0.1843 | Yes |
| 405 | 393186 | zgc:56235 |  |  | 28953 | -0.909 | -0.1815 | Yes |
| 406 | 566370 | slc25a6 |  |  | 29069 | -0.936 | -0.1816 | Yes |
| 407 | 337489 | plcd1b |  |  | 29137 | -0.954 | -0.1801 | Yes |
| 408 | 100536970 | drd5a |  |  | 29173 | -0.966 | -0.1776 | Yes |
| 409 | 494454 | fgf5 |  |  | 29186 | -0.971 | -0.1743 | Yes |
| 410 | 100000544 | ptger3 |  |  | 29255 | -0.991 | -0.1727 | Yes |
| 411 | novel.869 | - |  |  | 29307 | -1.002 | -0.1705 | Yes |
| 412 | 555482 | tacr3a |  |  | 29327 | -1.008 | -0.1673 | Yes |
| 413 | 692065 | fgf16 |  |  | 29415 | -1.037 | -0.1662 | Yes |
| 414 | 387297 | p2rx2 |  |  | 29433 | -1.043 | -0.1628 | Yes |
| 415 | 569706 | phkb |  |  | 29438 | -1.044 | -0.1590 | Yes |
| 416 | 558525 | atp2b3b |  |  | 29510 | -1.068 | -0.1572 | Yes |
| 417 | 564337 | ppp3r1a |  |  | 29604 | -1.097 | -0.1560 | Yes |
| 418 | 100134855\_1 | mst1rb |  |  | 29672 | -1.120 | -0.1539 | Yes |
| 419 | 259258 | p2rx4a |  |  | 29739 | -1.145 | -0.1517 | Yes |
| 420 | 378480 | pdgfaa |  |  | 29776 | -1.156 | -0.1484 | Yes |
| 421 | 100137117 | si:dkey-1h24.2 |  |  | 29950 | -1.224 | -0.1493 | Yes |
| 422 | 58133\_1 | ngfb |  |  | 29959 | -1.227 | -0.1449 | Yes |
| 423 | 568269 | htr6 |  |  | 30036 | -1.261 | -0.1426 | Yes |
| 424 | 556160 | gna14a |  |  | 30055 | -1.267 | -0.1384 | Yes |
| 425 | 100006675\_1 | prkcba |  |  | 30078 | -1.276 | -0.1342 | Yes |
| 426 | 58147\_1 | p2rx3a |  |  | 30081 | -1.278 | -0.1295 | Yes |
| 427 | 570493\_1 | grin2ab |  |  | 30114 | -1.296 | -0.1256 | Yes |
| 428 | 798565\_1 | ryr1a |  |  | 30119 | -1.299 | -0.1208 | Yes |
| 429 | 327067\_1 | slc25a4 |  |  | 30176 | -1.326 | -0.1176 | Yes |
| 430 | 565379\_1 | phkg1a |  |  | 30225 | -1.352 | -0.1140 | Yes |
| 431 | 336121\_1 | calm2a |  |  | 30324 | -1.400 | -0.1118 | Yes |
| 432 | 796537\_1 | kdrl |  |  | 30378 | -1.437 | -0.1081 | Yes |
| 433 | 561475 | plcd4b |  |  | 30447 | -1.475 | -0.1047 | Yes |
| 434 | 353247 | tnnc1a |  |  | 30580 | -1.568 | -0.1029 | Yes |
| 435 | 567289 | grpr |  |  | 30616 | -1.602 | -0.0980 | Yes |
| 436 | 569566 | LOC569566 |  |  | 30709 | -1.680 | -0.0946 | Yes |
| 437 | 566671 | orai1a |  |  | 30834 | -1.788 | -0.0918 | Yes |
| 438 | 100000981\_2 | htr2cl1 |  |  | 30877 | -1.840 | -0.0861 | Yes |
| 439 | 100005590\_1 | hrh2b |  |  | 30931 | -1.891 | -0.0807 | Yes |
| 440 | 492486\_1 | adra1bb |  |  | 31120 | -2.113 | -0.0787 | Yes |
| 441 | 100005049 | fgf1b |  |  | 31129 | -2.123 | -0.0709 | Yes |
| 442 | 560081 | ednrbb |  |  | 31234 | -2.295 | -0.0655 | Yes |
| 443 | novel.946 | - |  |  | 31344 | -2.513 | -0.0595 | Yes |
| 444 | 368717\_1 | itpka |  |  | 31364 | -2.556 | -0.0505 | Yes |
| 445 | 386856\_1 | pdgfra |  |  | 31415 | -2.695 | -0.0419 | Yes |
| 446 | 571431 | LOC571431 |  |  | 31422 | -2.716 | -0.0318 | Yes |
| 447 | 560564\_2 | pdgfc |  |  | 31473 | -2.838 | -0.0227 | Yes |
| 448 | 544664 | erbb3b |  |  | 31538 | -3.021 | -0.0133 | Yes |
| 449 | 565241\_2 | ppp3ccb |  |  | 31577 | -3.181 | -0.0025 | Yes |
| 450 | 387296\_1 | p2rx1 |  |  | 31605 | -3.275 | 0.0091 | Yes |
Table: GSEA details [plain text format]

  

Fig 2: CALCIUM\_SIGNALING\_PATHWAY(DRE04020)      
 Blue-Pink O' Gram in the Space of the Analyzed GeneSet

  

Fig 3: CALCIUM\_SIGNALING\_PATHWAY(DRE04020): Random ES distribution      
 Gene set null distribution of ES for **CALCIUM\_SIGNALING\_PATHWAY(DRE04020)**
